# A novel model of traumatic femoral head necrosis in rats developed by microsurgical technique

**DOI:** 10.1186/s12891-022-05289-7

**Published:** 2022-04-21

**Authors:** Yongxiang Lv, Xing Qiu, Gang Liu, Yunqing Wang, Yazhong Zhang, Wenbo Li, Ziqiang Zhu

**Affiliations:** 1 Department of Orthopedics, The Second Affiliated Hospital of Xuzhou Medical University, 221000 Xuzhou, Jiangsu China; 2 Department of Orthopedics, Affiliated Zhongshan Hospital of Dalian University, 116000 Dalian, Liaoning China

**Keywords:** Femoral head traumatic osteonecrosis, Animal model, Femoral neck fracture, Vasculature deprivation

## Abstract

**Background:**

Clinical angiography and vascular microperfusion confirmed that the femoral head retains blood supply after a collum femur fracture. However, no animal model accurately mimics this clinical situation. This study was performed to establish a rat model with retained viability of the femoral head and partial vasculature deprivation-induced traumatic caput femoris necrosis by surgery.

**Methods:**

Thirty rats were randomly divided into three groups (*n* = 10 per group): normal group, sham-operated group (Control), and ischemic osteonecrosis group. The femoral head of the normal group of rats underwent a gross anatomy study and microangiography to identify femoral head blood supply. Microsurgical techniques were used to cauterize the anterior-superior retinacular vessels to induce osteonecrosis. Hematoxylin and Eosin (H&E) staining were used for femoral head histologic assessment. Morphologic assessments of the deformity in and trabecular bone parameters of the femoral head epiphysis were performed using micro-CT.

**Results:**

The blood supply of the femoral head in rats primarily came from the anterior-superior, inferior, and posterior retinacular arteries. However, anterior-superior retinacular vasculature deprivation alone was sufficient in inducing femoral head osteonecrosis. H&E showed bone cell loss in nuclear staining, disorganized marrow, and trabecular structure. The bone volume (BV) decreased by 13% and 22% in the ischemic group after 5 and 10 weeks, respectively. The mean trabecular thickness (Tb.Th) decreased from 0.09 to 0.06 mm after 10 weeks. The trabecular spacing (Tb.Sp) increased from 0.03 to 0.05 mm after 5 weeks, and the epiphyseal height-to-diameter (H/D) ratio decreased.

**Conclusions:**

We developed an original and highly selective rat model that embodied femoral head traumatic osteonecrosis induced by surgical anterior-superior retinacular vasculature deprivation.

**Supplementary information:**

The online version contains supplementary material available at 10.1186/s12891-022-05289-7.

## Introduction

Osteonecrosis (ON), especially osteonecrosis of the femoral head (ONFH), is a common orthopedic disease. If left untreated, ON usually progresses to femoral head collapse with secondary osteoarthritis (OA) [1, 2]. Traumatic ONFH is an unsolved health problem worldwide [3]. The exact pathogenesis of femoral head traumatic osteonecrosis remains uncertain, but obstruction of the blood supply to the femoral head is the most important factor [4]. Every year, a large number of ONFH clinical cases due to hip trauma have been selected for arthroplasty [5]. To reduce the incidence of traumatic ON, it is essential to understand the disease mechanism of traumatic ON. Clinical trials of novel preventive measures and treatment modalities are hampered by the lack of suitable experimental animal models to mimic a full range of ONFH, from early histological necrotic changes to end-stage, including the structural and mechanical collapse of the femoral head [6].

Current studies have shown that animal models of ONFH are constructed mainly for surgical vascular deprivation induction [7], physical [8] and chemical [9] injury induction, and non-traumatic drug induction [10]. However, a drawback of these models is extensive necrosis of the femoral head, which cannot accurately mimic the clinical situation. Peled et al. did find that blood supply was not completely blocked in a model of vascular deprivation-induced ONFH in rats [11]. Clinical angiography and vascular microperfusion confirmed that after femoral neck fracture, the femoral head of most femoral neck fracture patients retains blood supply, with blood supply derived from the retinacular blood vessels through the epiphyseal arterial network structure above the epiphyseal scar and to the fine vascular branches within the femoral head. After femoral neck fracture, the retinacular arteries (especially the inferior retinacular arteries) supply blood to support viable femoral head tissue that could maintain femoral head bone marrow vitality [12].

Therefore, the new understanding of the vascular structure of the femoral head has led us to recognize the need for a novel rat model of traumatic ONFH. This is to investigate the mechanism of the repair process after avascular osteonecrosis, including the formation of blood vessels and bone tissue in traumatic ONFH. This model would represent a more realistic simulation of the disease progress and the performance of each stage. Currently, there are no specific studies on partial disruption of blood supply to the femoral head in a model of ischemic osteonecrosis in rats. The lack of such a model hinders a deeper understanding of the molecular pathways involved in the repair process of ischemic osteonecrosis. Therefore, this study established a rat model of traumatic ONFH induced by partial vascular deprivation, which preserved some femoral head viability.

## Animals and methods

### Rat

Thirty 20-week-old male Sprague Dawley rats were used in the present study. Experimental animals were housed in groups with a 12-h light/dark cycle and randomly provided food and water. Rats were randomly divided into 3 groups (10 rats per group): normal group, sham-operated group (Control), and ischemic osteonecrosis group. The study was approved by the Institutional Animal Care and Use Committee of the Affiliated Zhongshan Hospital of Dalian University. All methods were carried out in accordance with relevant guidelines and regulations.

## Normal arterial anatomy

Gross anatomy was performed on the rat’s femoral head’s vascular supply and obtained images to reconstruct into 3D imaging in the normal group of 10 rats (20 hips). In order to define the vascular anatomy around the rat’s femoral head, the microangiographic technique was applied based on previously described methods in human with some modifications [13]. After intravenous injection of 2% sodium pentobarbital (40 mg/kg) to anesthetize rats, we opened the thoracic cavity of each rat and severed the inferior vena cava (IVC) to drain the blood. We flushed the vasculature with 0.9% normal saline containing heparin sodium (100 U/mL) and injected it at a pressure of approximately 120 mmHg via a needle inserted in the left ventricle. The specimens were then pressurized fixed with a radiopaque contrast agent barium sulphate suspension (Blanc Fixe Micro, Sachtleben Chemie GmbH; mean grain size: 700 nm). The vascular supply in the femoral head was observed by gross anatomy. The femoral head was then removed for microscopic CT (micro-CT) scanning. Microangiography was performed with micro-CT to observe the blood vessels and the femoral head. 1024 CT images were acquired along the longitudinal axis with a resolution of 9.41 μm (high resolution setting). Reconstruction of the serial tomograms was done based on the raw data. By locating the vasculature around the femoral head, we were able to identify three vessel groups supplying the femoral head epiphysis: the anterior-superior, posterior, and inferior retinacular arteries.

## Induction of Ischemic ONFH

**Fig. 1:**
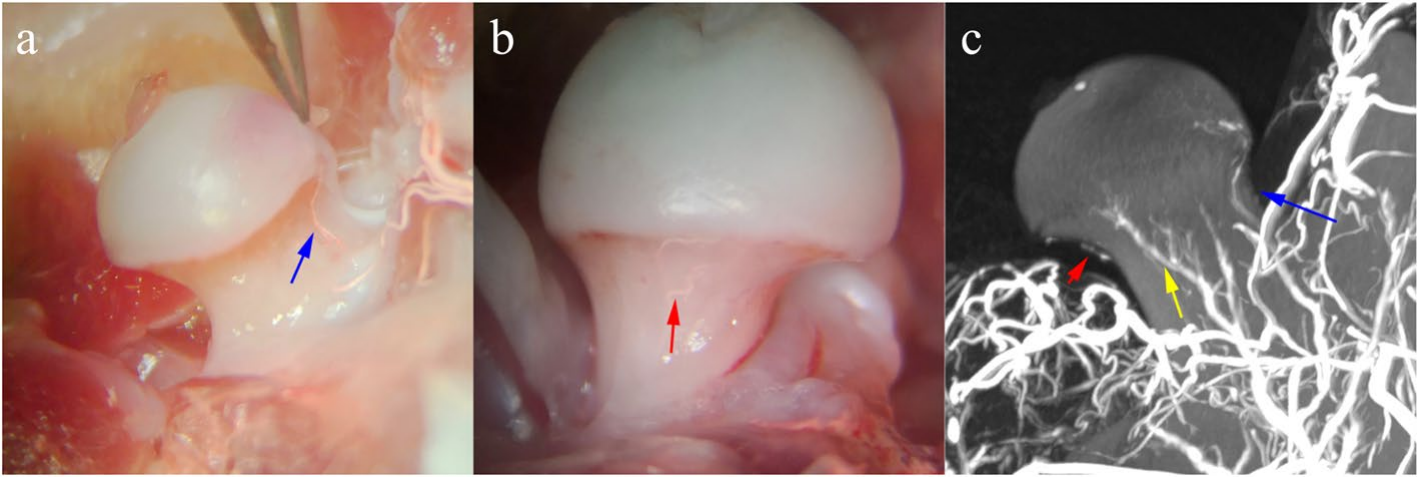
The normal rat femoral head blood supply. The rat femoral head is nourished by the anterior-superior, inferior and posterior retinacular arteries. The anterior-superior retinacular branches were the main stems of the femoral head epiphyseal artery. **a** The front view of the left hip showing the anterior-superior retinacular branches; **b** The inside view of the left hip showing the inferior retinacular branches; **c** The femoral head blood vessels perfusion and reconstruction showing the anterior-superior (Blue arrow), inferior (Red arrow) and posterior (Yellow arrow) retinacular arteries

The anterior-superior retinacular blood vessels supplying the femoral head were identified using a microscope and cauterized. Microsurgical instruments and bipolar coagulation were used to induce ischemic osteonecrosis (*n* = 10; Fig. 1a-b). The anterior-superior retinacular blood vessels were cauterized individually to induce effective local ischemia. The direct anterior approach for human total hip arthroplasty (THA) was used with some modifications, and the arthrotomy was closed with 9 − 0 sutures. Only the left femoral head underwent this surgery. In the control group (*n* = 10), superior retinacular blood vessels were exposed as the above description without cauterization. 5 rats in each group were sacrificed at each designated time after operation (after 5 and 10 weeks, respectively; *n* = 5 per time period), the femoral heads were collected.

### Histopathology analysis

The femoral heads were fixed with 10% formalin for 2 days before micro-CT performance. After scanning the specimen by micro-CT, they were decalcified with 10% ethylene diamine tetraacetic acid for 3 weeks. Femoral heads were embedded in paraffin and cut into 5 μm sections for hematoxylin and eosin (H&E) staining.

Histologic sections from femoral heads were examined to determine the number of empty lacunae and the femoral head structures. An observer unaware of the grouping performed measurements on three serial sections for qualitative analysis. Osteocyte lacunae were considered empty if there was no cell body or if they only contained pyknotic nuclei in the lacunae of the three serial sections.

### Micro-CT analysis

The femoral heads were scanned using a micro-CT system with settings as follows: 80 kV of energy and 500 µA of intensity. Trabecular bone parameters, including bone volume/total volume (BV/TV), trabecular thickness (Tb.Th), trabecular number (Tb.N), and trabecular spacing (Tb.Sp), were measured automatically. The following sections were analyzed on the two sacrificed groups mentioned above. We outlined each coronal section’s region of interest (trabecular bone in the femoral head epiphysis). Then, each specimen’s femoral heads were analyzed, and the mean value for each trabecular parameter was obtained. The height and width of the epiphysis in the mid-coronal plane on micro-CT images were measured to assess the deformity of the femoral head and evaluate the degree of femoral head collapse. The ratio of the femoral head epiphysial height to width was calculated in each specimen: the lower the height/width ratio, the more serious the deformity.

### Statistical analysis

Data were expressed as the mean ± standard deviation (SD). Micro-CT and quantitative histologic analyses were statistically analyzed with the independent sample t-test. A *P* value < 0.05 was considered statistically significant.

## Results

### Normal rat femoral head arterial anatomy

**Fig. 2:**
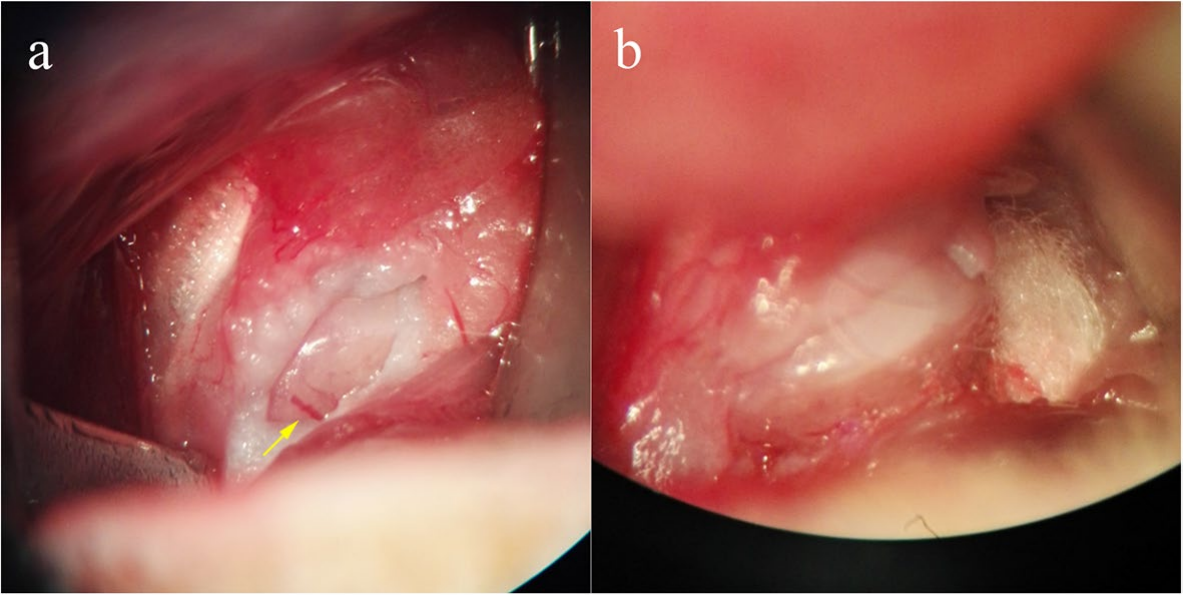
Vessel deprivation and creation of the rat femoral head osteonecrosis model. **a** is the anterior-superior retinacular blood vessels under the microscope. **b** shows the anterior-superior retinacular blood vessels after vessel deprivation

The rat femoral head received its primary supply of blood flowing from the anterior-superior, inferior, and posterior retinacular arteries. Compared with the anatomy of the blood supply of the human femoral head, in rats, the superior anterior artery located in the upper anterior part of the femoral neck and the lower retinaculum artery located in the medial part of the femoral neck both originated from the lateral femoral circumflex artery. The posterior retinacular originates from the obturator artery (Fig. 2a-c). The anterior-superior retinacular branches constitute the main stems of the epiphyseal artery after entering the femoral head. Its anatomic location is constant, with a larger diameter than the inferior and posterior retinacular arteries. The images of arteriography confirmed the existence of these vascular branches.

**Fig. 3:**
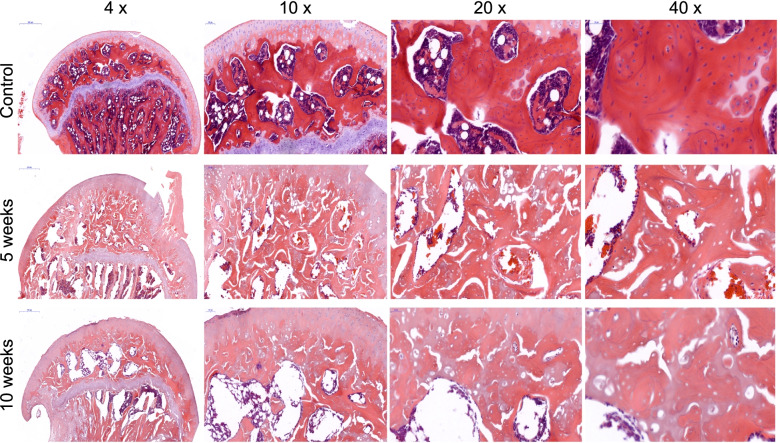
Hematoxylin and eosin staining of rat femoral heads. The deprivation of the anterior-superior retinacular blood vessels resulted in cell loss in most regions of the femoral head in terms of nuclear staining in the trabeculae and bone marrow; the structure was disorganized in the femoral head epiphysis after 5 and 10 weeks; however, the entire femoral head epiphysis did not appear completely necrotic. Chondral surfaces were not round and smooth in the ischemic group at week 10. In contrast, the chondral surface was round and smooth, and no cell death or bone structure disorganization manifested in the control group

Deprivation of the anterior-superior retinacular blood vessels that supply the femoral head epiphysis would result in mass cell death, but the femoral head epiphysis did not appear completely necrotic. The results of histologic sections qualitative assessment of the epiphysis showed a loss of nuclear staining of bone cells in most bone marrow cells, and bone marrow and trabeculae structures were disorganized at the fifth and tenth weeks post-surgery (Fig. 3). Sham-operated rats (Control group) femoral head showed no evidence of osteonecrosis (Fig. 3).

## Morphologic changes

**Fig. 4:**
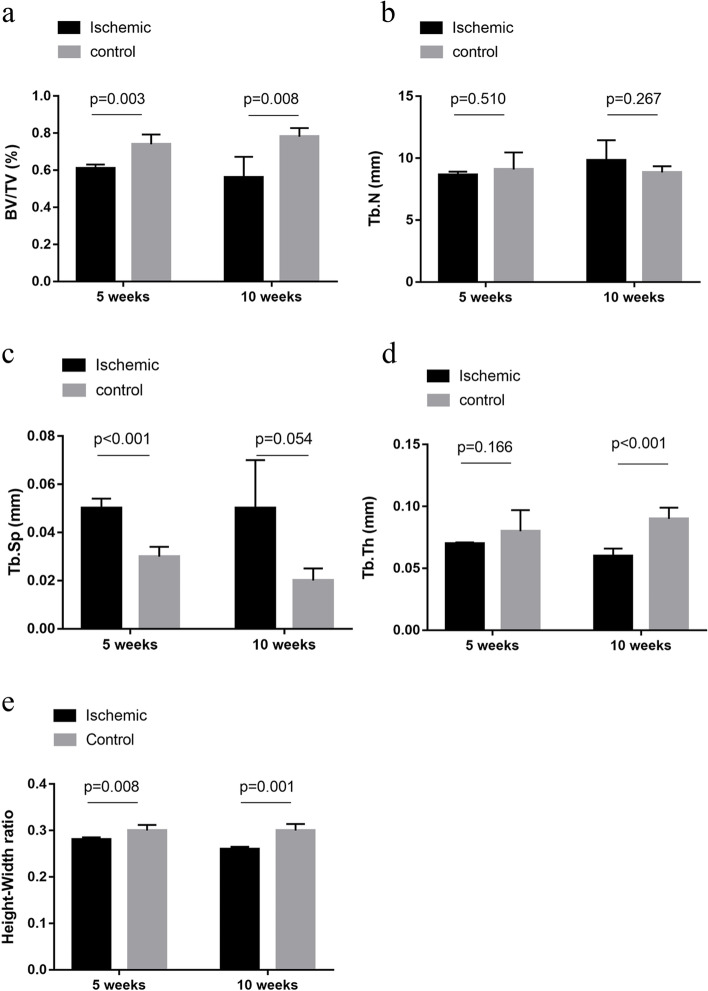
Morphological parameters of the femoral heads in the rats were analyzed by micro-CT. Micro-CT was used to assess bone volume percent (BV/TV %) (**a**), trabecular bone spacing (Tb.Sp)(**b**), trabecular number (Tb.N) (**c**), and trabecular thickness (Tb.Th) (**d**) in the femoral head epiphysis after induction of ischemic osteonecrosis. At 5 and 10 weeks, the ratio of height to diameter of epiphysis in the ischemic group was lower than that in the control group (**e**)

Through further analysis of the changes in femoral head trabecular parameters in each group of rats, compared with the control group, rats in the local ischemia group showed a notable decrease in BV/TV at weeks 5 and 10 (Fig. 4a). Tb.Th (Fig. 4d) decreased significantly at week 10 of local ischemia, while Tb.Sp (Fig. 4c) increased after week 5. Tb.N (Fig. 4b) was not significantly different between the two groups. At week 5 and week 10, the epiphyseal height to diameter (H/D) ratio in the ischemic group was lower than in the control group (Fig. 4e). There was no evidence of collapse shown in sham-operated rats. In addition, the shape of the femoral head epiphysis manifested a certain degree of deformity after 5 weeks, and the deterioration was found after 10 weeks by the height and diameter measurements of the epiphysis in mid-coronal micro-CT images (Supplementary Fig. 1a-c).

## Discussion

This study suggested that rats’ femoral heads take blood from the anterior-superior, inferior, and posterior retinacular arteries. Arising from the lateral circumflex femoral artery, the anterior-superior retinacular branches constitute the main stems of the epiphysis. The availability of a reliable rat model of traumatic ONFH facilitates the recognition of the mechanisms involved in the repair process after traumatic osteonecrosis. It facilitates the advancement of original treatment strategies to create simulations of vascular remodeling and femoral head healing. Nevertheless, there is currently no available rat model with partial disruption of the blood supply to the femoral head to induce traumatic femoral head osteonecrosis, which mimics the clinical situation of a femoral neck fracture.

We developed a reliable rat model of femoral head traumatic osteonecrosis. This model was developed by a systematic method that defined the vascular anatomy of the rat femoral head and then using microsurgical techniques to highly selective block merely the anterior-superior retinacular blood vessels that supply the femoral head. To our knowledge, this model is the first highly selective surgical model of traumatic femoral head osteonecrosis in rats. This study demonstrated that cauterization of the anterior-superior retinacular blood vessels was sufficient to cause extensive ischemic osteonecrosis in the femoral head epiphysis. All 10 rats suffered from extensive osteonecrosis in the femoral head epiphysis after cauterization, suggesting that it is a reliable model for inducing femoral head osteonecrosis. Since the epiphyseal plates of the rats never closed, a natural barrier was formed between the epiphysis and the metaphysis [14], which also divides the anastomotic vessels between the epiphysis and the metaphysis. The study of the three-dimensional anatomical structure of blood vessels in the human femoral head confirmed that the epiphysis and the metaphysis separated blood supply [12]. Therefore, the blood supply model of the rat femoral head is similar to that of the human adult femoral head.

The traditional rat model requires cutting off all the femoral head retinacular blood vessels and the round ligament of the femoral head, which causes femoral head dislocation inducing traumatic femoral head necrosis [15–17]. The anterior-superior retinacular blood vessels are responsible for the main blood supply to the femoral head; they are easy to identify and cauterize under a microscope applying microsurgical techniques. There was no need to destroy more retinacular blood vessels or cut off the ligaments of the femoral head with the risk of complications, including hip dislocation. In contrast, preserving inferior and posterior retinacular blood vessels can compensate for the circulation disruption after femoral head necrosis, one of this study’s major innovations. The traditional rat model of traumatic femoral head necrosis usually induces a 100% incidence of osteonecrosis, specifically in the femoral head [7, 15, 18]. In contrast, our model of surgically induced avascular osteonecrosis showed only massive areas of necrosis, and the osteonecrosis that occurred did not all happen in the femoral head epiphysis. The remaining viable tissue preserve repair and reconstruction ability of bone tissue.

In this study, micro-CT was used to assess the morphologic features of the trabeculae [19]. This high-resolution image technique allowed quantitative assessment of the whole femoral head epiphysis and the femoral head epiphysiss’ overall shape. This model showed femoral head deformity, as shown by the ratio of femoral head epiphyseal H/D in 5 and 10 weeks post-surgery [18, 20]. The trabeculae showed ivory-like changes in the femoral head epiphysis. The trabecular structure was disorganized for both ischemic groups. No evidence of collapse was shown in the sham-operated rats.

The development of bone deformity or collapse of the femoral head was a manifestation of the late stage of femoral head necrosis. While the necrosis continues to develop, it can lead to osteoarthritis, which has been found in some patients and large animal models of ischemic osteonecrosis. Nevertheless, it is less common in small animal models [21]. This feature can be used to evaluate potential treatments to prevent the development of femoral head osteonecrosis deformity. More observation time points to study the exact change in detail should be added in further studies.

Furthermore, the comparison of mean BV/TV percent results exposed a significant decrease in the ischemic groups. And Tb.Th and Tb.Sp mean also significantly decreased after 10 weeks and 5 weeks in the ischemic group. Whether the decrease in BV/TV, Tb.Th and Tb.Sp in the ischemic groups could be attributed to the imbalance of the resorption and formation of bone is not defined because micro-CT merely provides time-point measurements of the trabecular bone. These results suggest that the highly selective surgical model of traumatic femoral head osteonecrosis produced rat femoral head necrosis, bone loss, and femoral head deformity.

The study has limitations. (1) Only two time points of observation, 5 weeks and 10 weeks after surgery, were selected; (2) the number of rats in each group was too small; (3) Due to insufficient blood supply in this animal model, the process of bone necrosis, angiogenesis, new bone formation, bone structure change and reconstruction of femoral head necrosis could not be studied continuously. This study focused on the histological and micro-CT imaging studies of the femoral head that were carried out. In the future, relevant molecular pathways and potential pharmacological interventions should be explored.

## Conclusions

The anterior-superior, inferior and posterior retinacular arteries supply the rat femoral heads. We developed a reliable rat model of femoral head traumatic osteonecrosis through an approach to systematically define the vascular anatomy of the rat femoral head before using microsurgical techniques to perform highly selective disruption of only the anterior-superior retinacular blood vessels supplying the femoral head.

## Supplementary information

**Additional file 1: Fig. S1**MorphologicChanges of the femoral head on mid-coronal micro-CT images 

## Data Availability

The datasets supporting the conclusions of this article are included within the article.

## Abbreviations

BV: Bone volume
H/D: Height-to- diameter
H&E: Hematoxylin and eosin
OA: Osteoarthritis
ON: Osteonecrosis
ONFH: Osteonecrosis of femoral head
THA: Total hip arthroplasty
Tb.Sp: Trabecular spacing
Tb.Th: Trabecular thickness
SD: Standard deviation
