# Supplementary material for: A novel model of traumatic femoral head necrosis in rats developed by microsurgical technique
Source: BMC Musculoskelet Disord. 2022 Apr 21;23:374. doi: 10.1186/s12891-022-05289-7 (PMC9022312; doi:10.1186/s12891-022-05289-7)
Supplement: Supplementary file 1 — Additional file 1: Fig. S1MorphologicChanges of the femoral head on mid-coronal micro-CT images [file 12891_2022_5289_MOESM1_ESM.doc]

**
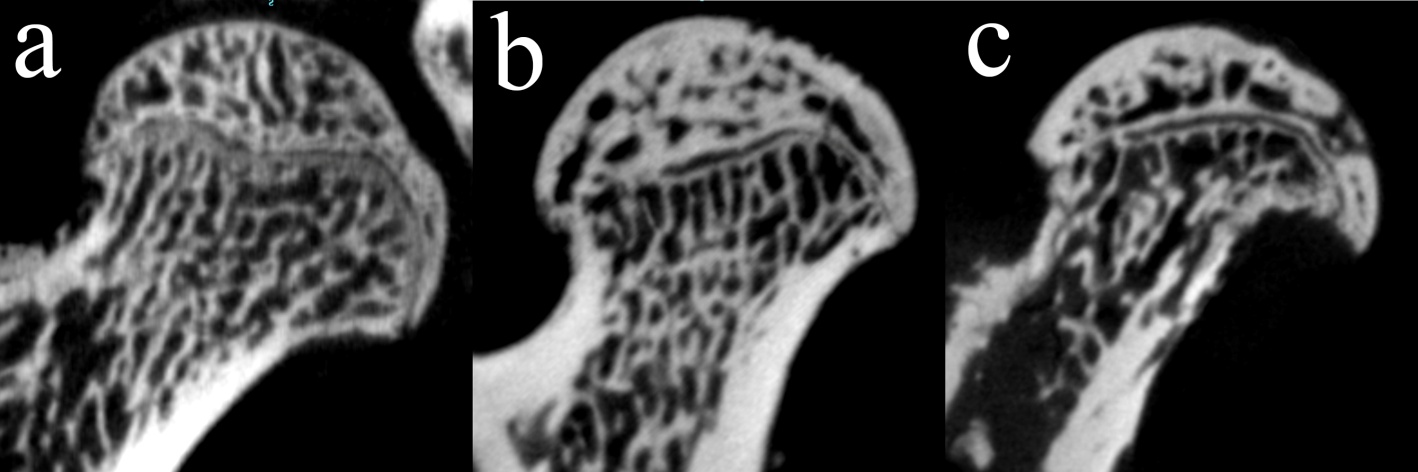
**

**Supplementary Figure 1.** **Morphologic Changes of the femoral head on mid-coronal micro-CT images.**

Mid-coronal micro-CT measurements of the height and diameter of the epiphysis were used to assess the degree of deformity in the shape of the femoral head epiphysis. The measurements showed a certain degree of deformity at 5 weeks, and at 10 weeks they indicate a more severe degree. The evidence of collapse didn't show up in rats with sham operation (a). In the femoral head epiphysis, there were trabeculae appearing with ivory-like changes and the trabecular structure was disorganized in the 5(b) and 10-weeks (c) ischemic groups.
